# The impact of nucleosome structure on CRISPR/Cas9 fidelity

**DOI:** 10.1093/nar/gkad021

**Published:** 2023-02-02

**Authors:** Christopher R Handelmann, Maria Tsompana, Ram Samudrala, Michael J Buck

**Affiliations:** Department of Biochemistry, Jacobs School of Medicine and Biomedical Sciences, State University of New York at Buffalo, Buffalo, NY 14203, USA; Department of Biomedical Informatics, Jacobs School of Medicine and Biomedical Sciences, State University of New York at Buffalo, Buffalo, NY 14203, USA; Department of Biochemistry, Jacobs School of Medicine and Biomedical Sciences, State University of New York at Buffalo, Buffalo, NY 14203, USA; Department of Biomedical Informatics, Jacobs School of Medicine and Biomedical Sciences, State University of New York at Buffalo, Buffalo, NY 14203, USA; Department of Biochemistry, Jacobs School of Medicine and Biomedical Sciences, State University of New York at Buffalo, Buffalo, NY 14203, USA; Department of Biomedical Informatics, Jacobs School of Medicine and Biomedical Sciences, State University of New York at Buffalo, Buffalo, NY 14203, USA

## Abstract

The clustered regularly interspaced short palindromic repeats (CRISPR) Cas system is a powerful tool that has the potential to become a therapeutic gene editor in the near future. Cas9 is the best studied CRISPR system and has been shown to have problems that restrict its use in therapeutic applications. Chromatin structure is a known impactor of Cas9 targeting and there is a gap in knowledge on Cas9’s efficacy when targeting such locations. To quantify at a single base pair resolution how chromatin inhibits on-target gene editing relative to off-target editing of exposed mismatching targets, we developed the gene editor mismatch nucleosome inhibition assay (GEMiNI-seq). GEMiNI-seq utilizes a library of nucleosome sequences to examine all target locations throughout nucleosomes in a single assay. The results from GEMiNI-seq revealed that the location of the protospacer-adjacent motif (PAM) sequence on the nucleosome edge drives the ability for Cas9 to access its target sequence. In addition, Cas9 had a higher affinity for exposed mismatched targets than on-target sequences within a nucleosome. Overall, our results show how chromatin structure impacts the fidelity of Cas9 to potential targets and highlight how targeting sequences with exposed PAMs could limit off-target gene editing, with such considerations improving Cas9 efficacy and resolving current limitations.

## INTRODUCTION

The specificity and simplicity of the clustered regularly interspersed palindromic repeats (CRISPR) Cas system make it a prevalent gene-editing tool. The power of the CRISPR Cas system has excellent therapeutic potential if key detriments can be overcome (1). One aspect limiting CRISPR Cas applications is the potential for Cas nuclease to cleave the genome at off-target locations (2,3). Determining the factors that cause the CRISPR Cas system to perform off-target modifications and mitigating these unintended results to produce the desired outcomes is crucial for further therapeutic applications. Further elucidating the well-established inability of the CRISPR Cas system to target various desirable sites in the genome due to chromatin structure and the impact on Cas editing fidelity can help address this deficiency (4,5).

The CRISPR Cas system evolved in bacteria and archaea as an adaptive immune system response to bacteriophage invasion, forming a family composed of members with greatly varying characteristics (6,7). The various CRISPR Cas systems are divided into Class 1, for systems that use multiple effector molecules, and Class 2, for systems that have only one effector molecule (8). Class 2 Cas systems, given the reduced number of components, have become the focus of engineering and development for use in gene editing, with the Cas9 nuclease being the first and most widely adopted (9). A prominently used version of Cas9 evolved in *Streptococcus pyogenes*, henceforth referred to as Cas9 (9,10).

The Cas9 system recognizes and cleaves a specific DNA target sequence through the interaction of a single guide RNA (sgRNA) with the Cas9 endonuclease. The sgRNA has two functional units: a scaffold region composed of three stem loops that complex with the Cas9 endonuclease and a targeting region containing a 20-nt sequence complementary to the DNA target sequence (11). Upon the sgRNA complexing with Cas9, the Cas9 endonuclease is reordered into an active conformation that interrogates DNA sequences (12). Cas9 initially searches the DNA for and binds the 3-bp NGG protospacer-adjacent motif (PAM) upstream of the 20-nt target sequence (13). Upon binding the PAM, the Cas9:sgRNA complex melts the DNA downstream of the PAM, and the sgRNA sequence invades, testing the potential DNA target for complementarity (14). If the DNA sequence has sufficient complementarity with the sgRNA sequence, then Cas9 cleaves the DNA target generating a blunt-ended double-strand break (15).

Previous studies have elucidated many vital factors underlying the Cas9 mechanism, such as the affinity for varying on-target sequence compositions (16), Cas9’s tolerance for mismatching sequences between the sgRNA and target DNA (5,17), and the effect of various eukaryotic chromatin states on Cas9 accessibility (18–20). Analyzing the impact of nucleosomes, the fundamental unit of chromatin, on Cas9 elucidated the factors impacting the dynamics of the more complex chromatin system. The nucleosome is composed of an ∼147-bp DNA sequence wound around a core histone octamer complex, making the DNA less accessible and thus impacting DNA interactions. To better define interactions, the locations within the nucleosomal DNA are defined relative to the center of the DNA wrap around histone octamer termed the dyad, with superhelical locations (SHLs) occurring at 10-bp intervals upstream (negative) and downstream (positive) from the dyad (SHL 0) (21). The nucleosomal DNA stops winding around the histone core a few bases after seventh SHL on either side of the dyad, forming the nucleosome edge. Further research into the impact of the nucleosome structure on Cas9 activity showed that occlusion of the PAM sequence within the nucleosome significantly increases protection from Cas9 cleavage (22), while exposure of the 20-nt target sequence beyond the nucleosome edge does not substantially impact Cas9 efficiency (23). However, these findings are limited to only a few target locations within a nucleosome and lack a direct quantification of Cas9’s affinity for occluded on-target compared to exposed off-target locations. Building further on these findings, our research utilizes an assay designed to test the impact of chromatin structure on a gene editor’s efficacy and applies it to determine the chromatin structure’s effect on Cas9 off-targeting.

To determine chromatin’s effect on Cas9 efficacy, we developed the gene editor mismatch nucleosome inhibition assay (GEMiNI-seq) as a modification of a transcription factor nucleosome binding assay (24,25). GEMiNI-seq uses a library of nucleosomes containing a Cas9 target and mismatch sequences at 1-bp resolution throughout a nucleosome. GEMiNI-seq can examine various factors simultaneously, utilizing the same sgRNA sequence to target the multiple nucleosome locations throughout the nucleosome structure, generating the most comprehensive mapping of Cas9 accessibility. In addition, we apply GEMiNI-seq to compare Cas9’s affinity for on-target and mismatch sequences relative to nucleosome structure, providing insight into the reasons for Cas9 off-targeting.

The results from GEMiNI-seq demonstrate that target sequences located within the nucleosome are protected from Cas9 digestion. This protection is dependent on the occlusion of the PAM sequence, with PAM sequences exposed outside the nucleosome having less protection. In addition, Cas9 efficacy is impacted by PAM sequence orientation in relation to the nucleosome. Crucially, our results also determine that Cas9 will preferentially target and digest a mismatch sequence with an exposed PAM sequence over an occluded on-target sequence. This preference for exposed mismatches over sterically blocked on-target sequences could drive the off-targeting prevalent in *in vivo* applications of Cas9 gene editing. Thus, our findings point to the importance of selecting Cas9 targets that are chromatin accessible and to avoid target sequences with chromatin accessible mismatches.

## MATERIALS AND METHODS

### GEMiNI-seq

A nucleosome library containing 7500 230-bp nucleosome sequences is designed and acquired from Agilent as a custom oligo library. The DNA sequences were amplified by PCR, column purified using Qiagen QIAquick PCR Purification Kit and quantified. Nucleosomes were formed from H2A/H2B dimer (160 pmol) and H3/H4 tetramer (80 pmol) from New England Biolabs (NEB), combined with 52.5 pmol of DNA, 1.68 M of sodium chloride and 1 µM of dithiothreitol diluted to 100 µl with TE (pH 8.0). The reaction is incubated at room temperature for 30 min and then transferred into a Slide-A-Lyzer MINI Dialysis Unit (10 000 MWCO, Thermo Scientific No. 69750). The dialysis unit is then successively placed on top of 1.2 ml of 1.0, 0.8 and 0.6 M sodium chloride for 2 h each at 4°C. The dialysis unit is then placed on top of 1.2 ml of TE (pH 8.0) overnight at 4°C. The nucleosome sample is collected and then purified with a sucrose gradient (24,25). Fresh 20% and 7% sucrose solutions are created and then a 7–20% sucrose gradient is prepared using a gradient mixer. The nucleosome sample is loaded on top of the sucrose gradient and then centrifuged on a SW41 rotor at 35 000 rpm for 18 h at 4°C. The sucrose gradient is fractionated and a portion of each fraction is run on a 4% native polyacrylamide gel to determine which contain properly formed nucleosomes. Nucleosome fractions are then concentrated and washed with TE (pH 8.0) in Amicon Ultra 0.5 ml 30k filters. The concentration of the nucleosome sample is then determined by qPCR.

sgRNA sequences were designed to target the DNA sequences tiled throughout the nucleosomal structure (Supplementary Table S1). The desired 20-nt target was flanked by the T7 promoter sequence and the 14-nt start for the RNA scaffold, following NEB protocol for target-specific oligo design. The DNA oligonucleotides were ordered from Integrated DNA Technologies, and the sgRNAs were produced using the NEB EnGen sgRNA Synthesis Kit (NEB #E3322). To ensure proper formation, the sgRNAs were run on a 10% denaturing TBE–urea gel, and the concentration of each was quantified. Each sample was diluted with nuclease-free water to a 300 nM working concentration. The Cas9 digestion is performed with equal amounts (300 nM) of CRISPR/Cas9 (NEB) and sgRNA sequences are incubated together in NEBuffer 3.1 at room temperature for 10 min. Nucleosomes (30 nM) are added and digested at 37°C for 30 min and then placed on ice. Proteinase K (20 µg) is added, and the reaction is incubated at room temperature for 10 min. The DNA is purified from the reaction and concentrated using a Qiagen MiniElute Purification Kit. Each digestion is replicated three times with an undigested control.

Illumina sequencing libraries were generated using two-step PCR, with 12 cycles of amplification for the first step using four sets of primers designed to offset sequence reads and dephase the libraries during Illumina sequencing (Supplementary Table S2). The second eight-cycle PCR barcodes the individual samples using Nextera Index primers for identification. The concentration of each sample was determined using the Invitrogen Quant-iT dsDNA Assay Kit, and equal amounts of each sample were pooled and sequenced on an Illumina NextSeq 2 × 150 at UB Genomics and Bioinformatics Core. The results from the sequencing were uploaded to the Sequence Read Archive under the accession number PRJNA868300.

### GEMiNI-seq analysis

The Illumina sequence reads were processed with a pipeline of applications to refine and identify the sequences present in the sample pool. The low-quality results at the 3′ end of the Illumina reads were removed by the Cutadapt tool, using a quality cutoff (−*q*) of 30 (26). The forward and reverse reads were merged using the Vsearch --fastq_mergepairs, only merging sequences with at least 20 overlapping nucleotides (--fastq_minovlen 20) and only allowing a maximum of two mismatches between merged sequences (--fastq_maxdiffs 2) (27). The primer sequences present at the end of the reads (Supplementary Table S2) were removed by Cutadapt. Any sequences >220 or <174 nt were removed, using the Cutadapt --maximum-length and --minimum-length functions, respectively. The reads were converted from fastq to fasta format using the FASTX-Toolkit FASTQ-to-FASTA converter function (http://hannonlab.cshl.edu/fastx_toolkit/index.html) before using Vsearch to identify each sequence in the nucleosome library (27). The search was performed by comparing the processed reads to the fasta formatted nucleosome library sequences (--dbmatched), rejecting sequence matches that have alignment lengths <150 nt (--mincols 150) or have <98.5% similarity (--id 0.985), and only reporting the hit with the highest percentage of identity (--top hits only).

The number of reads for each sequence present in a sample is compared relative to a native Widom 601 control sequence present within the nucleosome library, thus controlling for technical variability introduced by PCR, NGS library construction and NGS sequencing. ‘Protection from Cas9’ is defined as


(1) $$\begin{eqnarray*}&&{\rm Protection}\ {\rm from}\ {\rm Cas}9 \nonumber\\ &&= {{\rm log}}_2 \left( {\frac{{{{{\rm Reads}\ {{\rm Digested}}_{\rm N}}}/{{{\rm Reads}\ {{\rm Digested}}_{{\rm CON}}}}}}{{{{{\rm Reads}\ {{\rm Undigested}}_{\rm N}}}/{{{\rm Reads}\ {{\rm Undigested}}_{{\rm CON}}}}}}} \right),\nonumber\end{eqnarray*}$$

where Reads Digested_N_ is the number of uncleaved reads for a given nucleosomal target sequence in the sample pool after Cas9 digestion, Reads Digested_CON_ is the number of reads for the native Widom 601 control (Supplementary Figure S1) in the sample pool after Cas9 digestion, Reads Undigested_N_ is the number of reads for a given nucleosomal target sequence in the undigested sample pool and Reads Undigested_CON_ is the number of reads for the native Widom 601 control in the undigested sample pool. Each sample is then normalized by the *Z*-score with the mean and standard deviation defined from the nonspecific background sequences.

### MNase-seq on nucleosome library digestion

The nucleosome library (0.2 pmol/µl) is digested by MNase (0.05 U/µl) in nuclease digestion buffer (10 mM Tris–HCl, pH 8.0, 2 mM CaCl_2_) for a time course of 0 (no MNase used), 5 and 10 min at 37°C. After the defined incubation time, digestion was stopped (2% SDS, 40 mM EDTA). Proteinase K (16 µg) is added to each sample, and the reaction is incubated at 55°C for 1 h. The DNA is purified from the reaction and concentrated using a Qiagen MiniElute Purification Kit. The DNA concentration of each sample is determined by the Invitrogen Quant-iT dsDNA Assay Kit and equalized. Illumina sequencing libraries were generated using NEBNext Ultra II DNA library prep kit. Individual samples are multiplexed and sequenced on an Illumina MiSeq 2 × 150. MNase-seq sequencing results are quality filtered (*q* > 30) and adapter trimmed using Cutadapt (26). The quality reads are merged and mapped to the 7500 nucleosome library sequences using Vsearch (27). The read counts and end positions are used to determine MNase protection, which is a measurement of the percentage of reads for a specific nucleosome base pair location. MNase protection is calculated for each base pair as the ratio of base pair coverage/total reads for that specific nucleosome.

### Modeling Cas9 on the nucleosome

The structures for the Widom 601 nucleosome (28) and Cas9 (29) were retrieved from the Protein Data Bank (PDB) (30) (PDB identifiers 5OXV and 4UN3, respectively). The ChimeraX software (31) was used to load and edit the Widom 601 structure to a single nucleosome with 180 bp of DNA. The mutate bases function in the x3DNA software package (32) was used to change the Cas9 nontarget DNA strand (chain D), so the 12 DNA bases matched with the 12 DNA bases in chain J of the Widom 601 nucleosome, conserving 5′ to 3′ direction for all locations of interest at SHL 6.6 and SHL 7. The modeling of Cas9 in the opposite direction modified the nontarget DNA strand (chain D), so the 12 DNA bases matched with the 12 DNA bases in chain I of the Widow 601 nucleosome, conserving the 5′ to 3′ direction (SHL 7.4). The modified Cas9 structures were saved, and each was individually loaded into ChimeraX with the modified nucleosome structure. The Cas9 structure was positioned onto the nucleosome by the matchmaker function aligning the nontarget DNA chain in the nucleosome as the reference structure with the nontarget DNA chain in the Cas9 structure as the match structure, using the Needleman–Wunsch algorithm for sequence alignment. Steric hindrance between the Cas9 endonuclease and the nucleosome model for each location was determined and depicted by the Clashes function in ChimeraX. The number of clashes is determined between the Cas9 protein and the nucleosome structure up to SHL 5, excluding the DNA sequence being directly bound by Cas9 in quantifying the steric hindrance.

Each superimposed structure has hydrogen (AddH) and charges (addcharge) assigned through the Amber Tools force field within Chimera (33,34). Each structure is minimized using 600 steps of steepest descent followed by 60 steps of conjugate gradient, minimizing clashes within the structures. The minimized superimposed Cas protein is separated from the nucleosome, and each is run through BTTR (r·m·r), with Cas9 as the ligand and the nucleosome as the receptor, to calculate the favorability for Cas9 interacting at each location (35). BTTR is a knowledge-based discriminatory function that uses the atomic level radial distribution averages of all pairwise atom types to determine favorable interactions from incorrect ones. The BTTR program is run with the settings of -ref mean, -func radial, -comp reduced and -cutoff 12 (angstroms).

## RESULTS

### Nucleosome library design for Cas9 targeting

**Figure 1.**
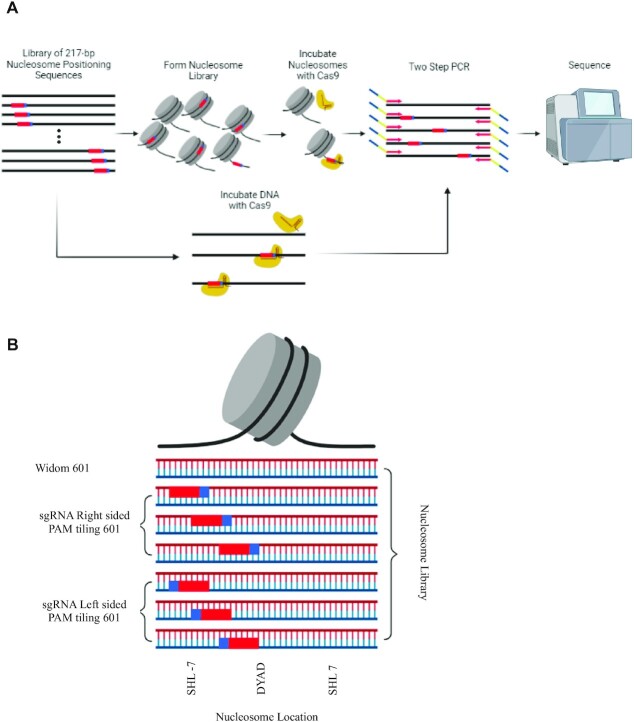
GEMiNI-seq method. (**A**) A 217-bp DNA library was designed containing a 20-bp target sequence and adjacent 3-bp PAM at every base pair position. The DNA library was formed into nucleosomes and purified forming a nucleosome library. Both the nucleosome and DNA library were separately incubated with a Cas9:sgRNA complex complementing the 20-bp target sequence. Samples are then indexed with two-step PCR and sequenced using an Illumina NextSeq. (**B**) A 20-bp DNA sequence and 3-bp PAM sequence (23 bp total length) are tiled at every base pair position through the Widom 601 nucleosome positioning sequence and into the linker. The location of the PAM site in the nucleosome is defined by the SHL from the nucleosome dyad. The PAM and target sequence are tiled in both a forward conformation (with the PAM sequence oriented to the 3′ of the target sequence) and a reverse conformation (with the PAM oriented to the 5′ of the target sequence).

To address the impact of nucleosome structure on Cas9 accessibility, we modified an approach described by Yu and Buck (24), to determine the targeting efficacy of the CRISPR Cas system within chromatin (Figure 1). Our new GEMiNI-seq methodology utilizes a nucleosome library with 7500 sequences. Each sequence is unique and allows the examination of Cas9’s specificity at all nucleosome positions with various mismatches. The nucleosome library is formed from the Widom 601 nucleosome positioning sequence, which is the best characterized nucleosome positioning sequence and can reproducibly position nucleosomes (36). A 23-bp sequence, composed of a 20-bp target sequence and adjacent 3-bp PAM sequence, is positioned at every nucleotide base through the nucleosome forming region and into the linker region until the full motif is exposed outside the histone octamer. Each sequence position replaces a new length of the Widom 601 sequence, generating a unique sequence in the nucleosome library. In this approach, we utilized a forward target sequence with the 20-bp target 5′ of the PAM, a reverse target with the 20-bp target 3′ of the PAM and a nontarget with a 18-bp target 5′ of the PAM (Supplementary Table S1). The nontarget, with a lack of sgRNA sequence complementation, acts as a negative control for Cas9 digestion. Targeting the same sequence eliminates any deviation in Cas9 digestion induced by varying sequence affinities and increases the efficiency of GEMiNI-seq (37).

The library of 230-bp DNA sequences was purchased from Agilent, amplified and formed into nucleosomes by salt gradient dialysis. Two sgRNAs are designed to complement either a forward or a reverse target sequence. After digestion, samples and controls are indexed and amplified by two-step PCR. Sequences undigested by Cas9 have both primer pairs allowing proper sequencing, while cleaved sequences will not have the two primer pairs, so they will not be present (38). Quantification of each sequence present in the sample pool creates an in-depth mapping of Cas9 protection throughout the nucleosome structure.

The library contains two different target sequences individually tiled across the Widom 601 nucleosome to elucidate any underlying impact of Cas9 orientation on access to the target sequence (Figure 1B). The forward sequence has the PAM sequence on the 3′ end (downstream) of the target sequence, and the reverse sequence has the PAM sequence on the 5′ end (upstream) of the target sequence. A negative control sequence containing a PAM but lacking a 20-bp target complementary to the sgRNAs is used to compare Cas9’s affinity for digesting the various target sequences. The further elucidation on the fidelity of Cas9 for mismatches relative to nucleosomal targets is achieved by designing sgRNAs complementary to the forward sequence except for alterations in the 19th or 20th base. The correlation of all the replicates shows a high similarity within replicates of the same digestion, and a similarity between digestions of the same substrate with similar sgRNAs (Supplementary Figure S2).

### Nucleosomes protect target sequences from Cas9 digestion

**Figure 2.**
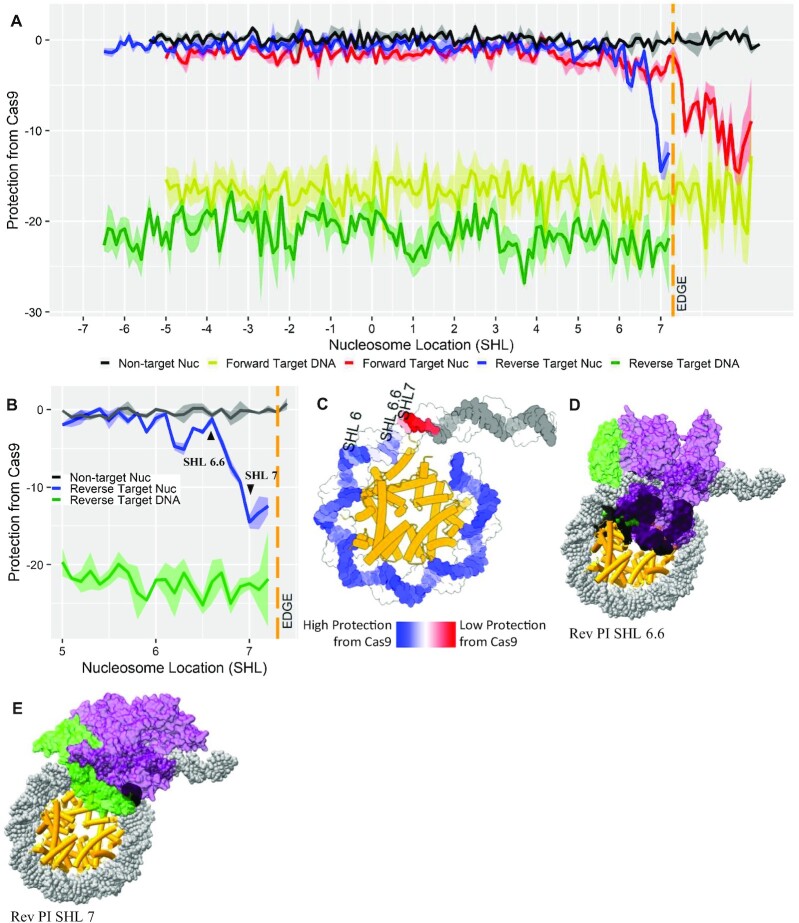
Nucleosomes protect potential targets from Cas9 digestion. (**A**) Comparison of the protection of a nontarget, forward target and reverse target at every base pair within the nucleosome structure and the protection for the same forward and reverse targets at every base pair in naked DNA. (**B**) The protection of the reverse target sequence, with the last point of maximum protection (SHL 6.6) and the first point of least protection (SHL 7) indicated. (**C**) The nucleosome structure has the protection from Cas9 values depicted as a heatmap. Superimposition of the Cas9 endonuclease relative to the target sequence within the nucleosome structure at (**D**) SHL 6.6 and (**E**) SHL 7. The ribbons within the line graphs are error of the mean for protection from Cas9 values.

To determine the protection the histone octamer provides the Cas9 target sequences, we incubated the nucleosome library with a Cas9:sgRNA complex complementary to either the forward or reverse target sequences. Quantification of the relative numbers of each undigested sequence generates a map of protection for the nucleosomal and linker DNA. The same digestion is performed on the naked DNA library as a control for sequence position effect. Both naked targets show substantial digestion, with digestion of the reverse target being greater than the forward target (Figure 2A), which may result from the forward and reverse target sequences having different nucleotide compositions (Supplementary Figure S1) (39,40).

The majority of sequences in our nucleosome library are nonspecific to these Cas9 digestions and show no cleavage throughout their entire sequence. Both the forward and reverse target sequences show high protection from Cas9 digestion when located within the nucleosome structure. The amount of protection is equivalent to the protection for the nontarget and is regardless of the location of the PAM sequence or its orientation relative to the histone octamer. The forward and reverse targets both maintain a high level of protection until the PAM sequence nears the edge of the nucleosome, at which point both sequences have a loss in protection from Cas9 digestion. Protection decreases for both the forward and reverse target sequences located at the edge of the nucleosome and within the linker, with the amount of protection for these targets nearing the values of naked DNA. The reverse sequence has the PAM sequence upstream of the target, so the target exits the nucleosome first, yet the protection only decreases when the PAM sequence nears the edge of the nucleosome making the whole target accessible. The forward sequence has the PAM sequence downstream of the target sequence, so the PAM exits the nucleosome first, but the forward target is protected for an additional 6 bp from where the reverse target protection decreases (Figure 2A). The reduced accessibility of the Cas9:sgRNA complex to the entire forward target sequence compared to the fully exposed reverse target sequence likely drives this difference in protection.

To determine whether the last local maximum protection (Figure 2B, SHL 6.6) and the first local minimum protection (Figure 2B, SHL 7) spanning the drastic change in protection at the nucleosome edge relate to less or more sterically inhibited orientations of Cas9, we modeled Cas9 orientation when accessing these PAM sequences. The Cas9 structure was superimposed onto both locations at the nucleosome edge, aligning the nontarget DNA strand in both structures to maintain proper orientation. The superimposition of Cas9 onto the reverse target PAM at SHL 6.6 shows Cas9 being orientated into the nucleosome structure in an unfavorable conformation, with 4544 clashes occurring between the two structures (Figure 2C and Supplementary Figure S3). In contrast, 4-nt downstream at SHL 7, the orientation of Cas9 endonuclease on the reverse target PAM sequence is positioned opposite to the nucleosome structure, making the PAM sequence more accessible with only 415 clashes (Figure 2D and Supplementary Figure S3). The change in the orientation of Cas9 to the nucleosome when accessing the two various sequences could induce the fluctuations in protection seen once the PAM domain becomes exposed, while PAM sequences located deeper within the nucleosome have Cas9 access blocked by the histone octamer (22,41).

### Nucleosome library accessibility defined with MNase-seq

**Figure 3.**
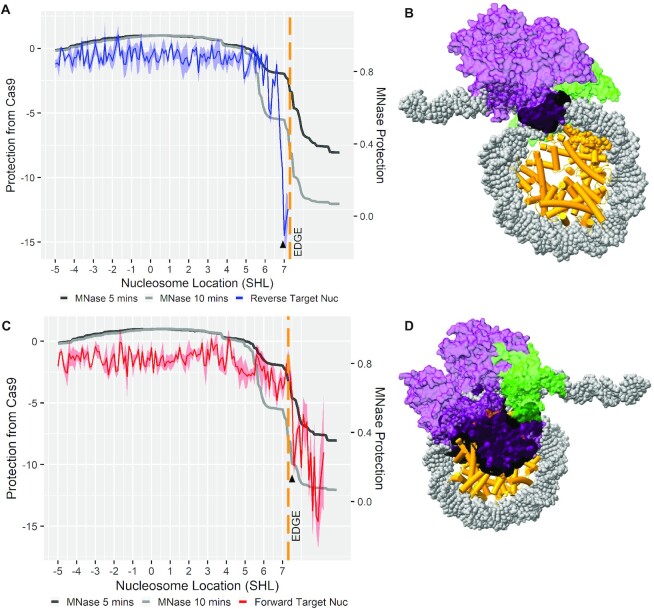
MNase accessibility of nucleosome library compared to Cas9 accessibility. (**A**) Protection of the reverse target sequence compared with the MNase accessibility. The ‘Δ’ indicates the first substantial drop in target sequence protection from Cas9 digestion (SHL 7). (**B**) The Cas9 endonuclease structure is imposed onto nucleosome structure at the ‘Δ’ position in panel (A). (**C**) Protection of the forward target sequence with the MNase time course. The ‘Δ’ indicates the first substantial drop in target sequence protection from Cas9 digestion (SHL 7.6). (**D**) The Cas9 endonuclease structure is imposed onto the nucleosome structure at the ‘Δ’ position in panel (C). In panels (B) and (D), the green indicates the Cas9 PI domain, purple is the remaining Cas9 structure and the black shading indicates clashes between the PI domain and the nucleosome structure. For panels (A) and (C), the ribbons within the line graphs are standard error of the mean for protection from Cas9 values.

To determine how the protection supplied by the nucleosome from Cas9 targeting compares to the accessibility of the nucleosome structure, we compared the footprint of the Widom 601 nucleosome generated by incubation with the nonspecific endo-exonuclease micrococcal nuclease (MNase) to the protection values for the forward and reverse target sequences. Digesting the nucleosome with MNase removes the accessible DNA, while the inaccessible DNA regions bound to the histone octamer proteins remain intact. A time course of digestion (Figure 3A and C) shows MNase readily digesting most nucleosomal DNA up to around SHL 7, at which point ∼55% of the DNA remains undigested by MNase and a further decrease in DNA digestion at SHL 5.5 with ∼85% of the DNA sequences remaining undigested. The nucleosome footprint corresponds with the loss of protection observed for both the forward and reverse targets immediately downstream of this location.

Cas9 is able to access the reverse target sequence at SHL 7 and the forward target at SHL 7.6 (Figure 3A and C). The location and orientation of the target sequence relative to the nucleosome structure appear to drive the observed change in Cas9 accessibility. The 20-nt reverse target sequence located downstream of the PAM sequence allows Cas9 to readily target the complete sequence when the PAM is located in a more accessible region of the nucleosome. When the PAM of the reverse target is located at SHL 7, there is a drop in the amount of protection from Cas9 (−14.49). The drop in the value of protection from Cas9 is located at the edge of the MNase footprint for the nucleosome and is 15 nt downstream of the stronger MNase barrier around SHL 5.5 (Figure 3A). In comparison, there is higher protection of the forward target from Cas9 digestion when the PAM is located at SHL 7 (−3.85), showing that the target sequence orientation upstream or downstream of the PAM sequence is impacting Cas9 digestion. To determine the amount of steric hindrance Cas9 would encounter while accessing this location, we imposed the Cas9 structure over the reverse target sequence based on PAM location at SHL 7, which results in 415 clashes occurring between the two structures (Figure 3B and Supplementary Figure S3). Imposing Cas9 onto another reverse target location 10 bp closer to the dyad at SHL 6 results in 2484 clashes (Supplementary Figure S3), indicating that the reverse targets closer to the dyad are also unfavorable. Imposing the Cas9 structure onto the forward target sequence at SHL 7 results in 9581 clashes (Supplementary Figure S3), showing the unfavorable orientation of Cas9 for the forward target at SHL 7.

When the forward target PAM is located at SHL 7.6, there is a drop in the value of protection from Cas9 (−10.07), 6 nt downstream of the nucleosome barrier around SHL 7 (Figure 3C and Supplementary Figure S3). With the center of the PAM motif at SHL 7.6, the 20-nt forward target sequence extends to SHL 5.5, where the stronger MNase barrier also resides. Forward target sequences located closer to the nucleosome dyad will have the targeting sequence obscured within the more inaccessible nucleosomal location. Imposing the Cas9 structure onto the PAM location at SHL 7.6 and oriented with the forward target sequence shows a high number of clashes (7409) (Figure 3D and Supplementary Figure S3). The increased number of clashes for the forward target sequence at SHL 7.6 (7409) compared to the number of clashes for the reverse target sequence at SHL 7 (415) matches the higher protection from Cas9 value the forward target sequence has at SHL 7.6 (−10.07) compared to the reverse target sequence’s lower protection from Cas9 value (−14.49) at SHL 7 (Figure 3). To further investigate the structural interactions of Cas9 with the nucleosome, we imposed only Cas9’s PAM interfacing (PI) domain onto the reverse target PAM at SHL 7 and onto the forward target at SHL 7.6. The PI domain had minimal clashes with the nucleosome structure when accessing the reverse target PAM at SHL 7 (0 clashes) or the forward target PAM at SHL 7.6 (119 clashes), showing that both PAM locations are relatively accessible (Supplementary Figure S3).

The difference in Cas9 access to the forward and reverse targets is thus from target sequence orientation varying Cas9’s interaction with the nucleosome structure. While Cas9 is being more hindered in accessing the complete forward target within the nucleosome, Cas9 is still accessing and digesting the full target sequence, possibly through a combination of nucleosome breathing and Cas9 acting as a Brownian ratchet to compete the DNA off from the nucleosome (42,43). When the forward sgRNA target is further upstream, with the PAM at SHL 7, the stronger binding of the DNA to the nucleosome, around SHL 5.5, blocks Cas9 from accessing the complete forward target. In comparison, the reverse sgRNA target with the PAM sequence at the same position is accessed and digested Cas9 showing that the impact is not driven by PAM accessibility.

### Protection of off-target sequences from Cas9 digestion

**Figure 4.**
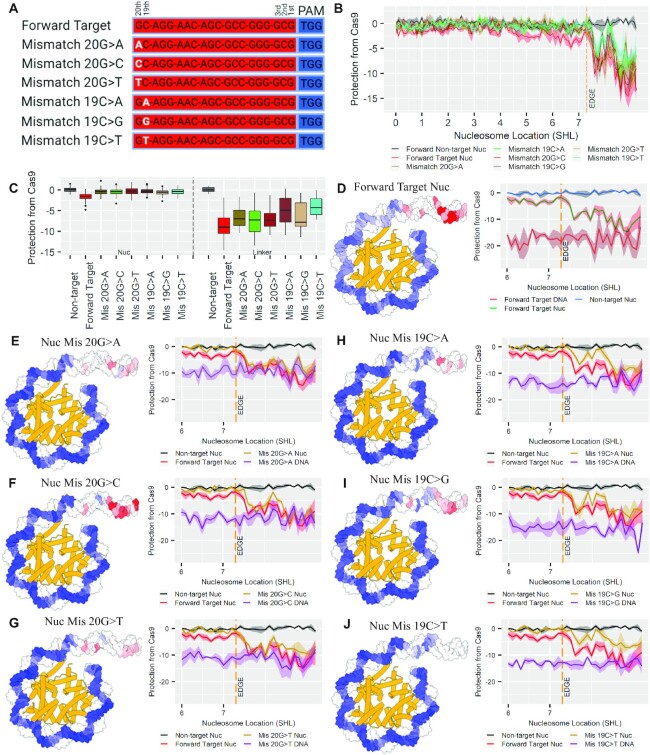
Protection of mismatch target sequences from Cas9 digestion. (**A**) The sgRNAs designed to mismatch at the 20th and 19th bases in the forward target sequence. (**B**) Comparison of the protection for the nontarget, forward target and the various mismatches for the forward target sequence throughout the whole nucleosome. (**C**) Box and whisker plot comparing the amount of protection for the forward target and nontarget to the mismatch targets in both the nucleosome (SHL-5 to edge) and the linker (edge to end). (**D**–**J**) On the left is a nucleosome structure with the amount of protection from Cas9 depicted as a heatmap on the Watson strand. On the right is a line graph comparing the Cas9 protection for the nontarget and forward target (D) compared to mismatches for the 20th (E–G) and 19th (H–J) bases centered on the nucleosome’s edge. The ribbons within the line graphs are error of the mean for protection from Cas9 values.

To elucidate the impact of targeting a mismatching sequence throughout the nucleosomal structure, we designed sgRNA sequences with mismatching nucleotides in the 20th and 19th bases, distal to the PAM, of the forward target sequence. Six mismatch sgRNAs were generated, with all three alternative nucleotide bases substituted into both locations (Figure 4A). The impact of the nucleosome structure on the protection for the mismatching sequences is similar to the on-target sequences, with both on-target and mismatching targets located in the nucleosome being highly protected from Cas9 digestion and targets located outside the nucleosome structure having substantially lower protection from Cas9 values (Figure 4B). The location of the mismatch within the target sequence impacts Cas9’s affinity for the given sequence, with the mismatch at the 19th base offering more protection from Cas9 digestion than the mismatch at the 20th base, which is more protected than the on-target sequence (Figure 4C). The impact of mismatches is seen both in the linker region and within the nucleosome, showing that Cas9 is digesting nucleosomal DNA and this digestion is impacted by sequence fidelity (Supplementary Figure S4). Our results corroborate previous findings that mismatches closer to the PAM sequence decrease Cas9 affinity and digestion of the target (44). All mismatch targets located within the linker have protection from Cas9 values below the on-target sequence located within the nucleosome (Figure 4C). The lower protection of the exposed mismatch target sequences shows that Cas9 can better access and digest the exposed mismatch targets, and this direct comparison shows that Cas9 will more readily digest an exposed mismatch over an on-target sequence obscured within a nucleosome. The mismatching targets show decreased digestion at the nucleosome edges relative to the on-target (Figure 4D–J), showing a compounding impact on Cas9 digestion similar to previously reported results (20). The target locations within the linker region also have a similar pattern of protection from Cas9, with high and low protection values appearing in the same structural locations for both the forward target and the mismatches (Figure 4D–J). This observed patterning may result from the steric hindrance between Cas9 and the nucleosome as observed in a previous study by Makasheva *et al.* (22). Target locations distal to the nucleosome structure have the lowest protection from Cas9, showing that Cas9 accessibility to targets nearby nucleosomes can still be impacted by the nucleosome structure.

## DISCUSSION

To determine how chromatin structure impacts the efficacy of gene editors, we developed the GEMiNI-seq technique to compare target accessibility within nucleosomes and naked DNA. GEMiNI-seq allowed for a direct comparison of Cas9 accessibility and digestion of a target sequence at every position within a nucleosome in a single experiment with the same guide RNA eliminating guide-specific effects. Previous research has shown that nucleosomes hinder large molecular machinery from accessing DNA, such as with base excision repair (45–47). This problem is present in the therapeutic application of Cas9 for accessing and digesting targets within eukaryotic organisms (18–19,22–23,48). Our experiments support these results, with the nucleosome protecting target sequences from Cas9 endonuclease digestion with a level of nucleosome protection similar to a nontarget sequence lacking any sgRNA complementation.

A previous study by Hinz *et al.* (23) shows that PAM exposure is critical to Cas9 accessing its target sequence. Our results expanded on these findings by examining all possible variations in PAM sequence exposure relative to the nucleosome edge, adding substantial detail to the impact of PAM exposure. As seen with the forward target, when the PAM sequence is located at the edge of the nucleosome, Cas9 can digest the targeting sequence, even while it is buried within the nucleosome. However, the drop in the protection from Cas9 value for the forward target at SHL 7.6 is both higher and further downstream than the drop for the reverse target value at SHL 7. Previous studies show that both access to the PAM sequence and completion of R-loop formation are necessary for target digestion (22). The drop in protection from Cas9 for the forward and reverse targets at SHLs 7.6 and 7, respectively, occurs when both PAM accessibility and further R-loop formation are able to proceed. Since the reverse target is downstream of the PAM sequence, there is less inhibition to R-loop formation and digestion once the PAM sequence is accessible. The forward target positioned upstream of the PAM makes Cas9 contend with hindrance from the nucleosome while proceeding through R-loop formation, and thus decreases the digestion of the forward target. Cas9 is able to compete with the nucleosome for access to the forward target, possibly by acting as a Brownian ratchet (14).

Several studies show that Cas9 has less tolerance for DNA sequences with mismatches compared to sequence with complete complementarity (on-target sequences) and the mismatching base was best tolerated distal to the PAM, with decreasing tolerance as the mismatching base approached the PAM sequence (20,22,17,49,50). Our results showing a reduced tolerance for mismatches at base 19 compared to mismatches at base 20 in the exposed linker region are consistent with these findings. The protection offered by mismatches in the linker region, though variable, is less than the amount of protection the nucleosome provided to both the mismatches and the on-target sequences from Cas9 digestion. The difference in protection shows that when a mismatch sequence has an accessible PAM sequence, it is more likely to be targeted by Cas9 than a completely complementary sequence with a PAM sequence occluded within the nucleosome. This preference for exposed mismatches over sterically blocked on-target sequences could drive the off-targeting prevalent in applications of Cas9 gene editing. A previous study by Hinz *et al.* (20) shows that single-base mismatches have a greater impact on decreasing Cas9 digestion within the nucleosome than in naked DNA, with this impact increasing as the mismatch approaches the PAM. GEMiNI-seq corroborates mismatches decreasing Cas9 digestion within the nucleosome and into the linker.

Cas9’s preference for exposed mismatching targets over on-target sequences buried within nucleosomes clearly shows the impact of chromatin structure on the fidelity of Cas9 gene editing. Over 90% of the eukaryotic genome is inaccessible, with only a low percentage being in an active and open state (51). Our results show that it is crucial to target Cas9 gene editing to regions with open chromatin locations to optimize the desired result and reduce the possibility of undesirable off-targeting. The accessible regions within the genome are dynamic and cell type dependent (52). Mapping the chromatin structures of the desired cell population is possible with various techniques, such as MNase-seq, DNase-seq and ATAC-seq, and should be a prerequisite for future therapeutic gene editing. Gene editing is currently limited in the scope of therapeutic applications, but the proper selection of targets within accessible regions of the genome will improve the application of Cas9 editing, making the potential for therapeutic applications safer and more attainable.

Previous research shows that Cas9 targeting is impacted by chromatin structures *in vivo* (4–5,53). Our current results utilize the Widom 601 nucleosome positioning sequence (Supplementary Figure S1) to take advantage of the well-characterized positioning, stability and structural data available for this nucleosome. Widom 601 binds firmly around the histone octamer, and thus may not accurately represent the plasticity present in all nucleosomes. Incorporating other nucleosome sequences into future library designs will allow for further elucidation of how Cas9 interacts with chromatin structure for various *in vivo* applications and generate further understanding and guidance in applying Cas9 as a gene-editing therapeutic.

Cas9 experimental design has been informed through tools that select the optimal sequence to be used. The tools are designed to select ideal targets based on the aggregation of Cas9 digestion results from a multitude of studies showing ideal sgRNA sequence composition, RNA secondary structure and the impact of potential target mismatches to reduce off-targeting (16,54–57). The number of tools for sgRNA design is diverse and expanding, each with different algorithm designs for addressing the selection of Cas9 targets and different data points informing the tool selection (54,58). The impact of chromatin on the efficacy of various sgRNAs has been assessed within tool design, though chromatin state remains a nebulous aspect in target selection (16,56,58). The results from GEMiNI-seq may be used to inform tools on the selection of targets. Supplying tools with the quantified target digestion given chromatin structure, mismatch tolerance and sequence can yield a generalizable rule set that could select sgRNA targets based on the knowledge of these characteristics.

## DATA AVAILABILITY

The datasets generated and/or analyzed during the current study are available in the Sequence Read Archive under the accession number PRJNA868300.

## Supplementary Material

gkad021_Supplemental_File

## References

[1] Savic N. , Schwank G. Advances in therapeutic CRISPR/Cas9 genome editing. Transl. Res. 2016; 168:15–21. 26470680 10.1016/j.trsl.2015.09.008

[2] Pattanayak V. , Lin S., Guilinger J.P., Ma E., Doudna J.A., Liu D.R. High-throughput profiling of off-target DNA cleavage reveals RNA-programmed Cas9 nuclease specificity. Nat. Biotechnol. 2013; 31:839–843. 23934178 10.1038/nbt.2673 PMC3782611

[3] Ma Y. , Zhang L., Huang X. Genome modification by CRISPR/Cas9. FEBS J. 2014; 281:5186–5193. 25315507 10.1111/febs.13110

[4] Wu X. , Scott D.A., Kriz A.J., Chiu A.C., Hsu P.D., Dadon D.B., Cheng A.W., Trevino A.E., Konermann S., Chen S. et al. Genome-wide binding of the CRISPR endonuclease Cas9 in mammalian cells. Nat. Biotechnol. 2014; 32:670–676. 24752079 10.1038/nbt.2889 PMC4145672

[5] Kuscu C. , Arslan S., Singh R., Thorpe J., Adli M. Genome-wide analysis reveals characteristics of off-target sites bound by the Cas9 endonuclease. Nat. Biotechnol. 2014; 32:677–683. 24837660 10.1038/nbt.2916

[6] Horvath P. , Barrangou R. CRISPR/Cas, the immune system of bacteria and archaea. Science. 2010; 327:167–170. 20056882 10.1126/science.1179555

[7] Makarova K.S. , Koonin E.V. Annotation and classification of CRISPR–Cas systems. Methods Mol. Biol. 2015; 1311:47–75. 25981466 10.1007/978-1-4939-2687-9_4 PMC5901762

[8] Makarova K.S. , Wolf Y.I., Alkhnbashi O.S., Costa F., Shah S.A., Saunders S.J., Barrangou R., Brouns S.J., Charpentier E., Haft D.H. et al. An updated evolutionary classification of CRISPR–Cas systems. Nat. Rev. Microbiol. 2015; 13:722–736. 26411297 10.1038/nrmicro3569 PMC5426118

[9] Tang Y. , Fu Y. Class 2 CRISPR/Cas: an expanding biotechnology toolbox for and beyond genome editing. Cell Biosci. 2018; 8:59. 30459943 10.1186/s13578-018-0255-x PMC6233275

[10] Thurtle-Schmidt D.M. , Lo T.W. Molecular biology at the cutting edge: a review on CRISPR/CAS9 gene editing for undergraduates. Biochem. Mol. Biol. Educ. 2018; 46:195–205. 29381252 10.1002/bmb.21108 PMC5901406

[11] Charpentier E. , Doudna J.A. Biotechnology: rewriting a genome. Nature. 2013; 495:50–51. 23467164 10.1038/495050a

[12] Jiang F. , Doudna J.A. CRISPR–Cas9 structures and mechanisms. Annu. Rev. Biophys. 2017; 46:505–529. 28375731 10.1146/annurev-biophys-062215-010822

[13] Mojica F.J.M. , Diez-Villasenor C., Garcia-Martinez J., Almendros C. Short motif sequences determine the targets of the prokaryotic CRISPR defence system. Microbiology. 2009; 155:733–740. 19246744 10.1099/mic.0.023960-0

[14] Sternberg S.H. , Redding S., Jinek M., Greene E.C., Doudna J.A. DNA interrogation by the CRISPR RNA-guided endonuclease Cas9. Nature. 2014; 507:62–67. 24476820 10.1038/nature13011 PMC4106473

[15] Wiedenheft B. , van Duijn E., Bultema J.B., Waghmare S.P., Zhou K., Barendregt A., Westphal W., Heck A.J., Boekema E.J., Dickman M.J. et al. RNA-guided complex from a bacterial immune system enhances target recognition through seed sequence interactions. Proc. Natl Acad. Sci. U.S.A. 2011; 108:10092–10097. 21536913 10.1073/pnas.1102716108 PMC3121849

[16] Moreno-Mateos M.A. , Vejnar C.E., Beaudoin J.D., Fernandez J.P., Mis E.K., Khokha M.K., Giraldez A.J. CRISPRscan: designing highly efficient sgRNAs for CRISPR–Cas9 targeting *in vivo*. Nat. Methods. 2015; 12:982–988. 26322839 10.1038/nmeth.3543 PMC4589495

[17] Fu Y. , Foden J.A., Khayter C., Maeder M.L., Reyon D., Joung J.K., Sander J.D. High-frequency off-target mutagenesis induced by CRISPR–Cas nucleases in human cells. Nat. Biotechnol. 2013; 31:822–826. 23792628 10.1038/nbt.2623 PMC3773023

[18] Isaac R.S. , Jiang F., Doudna J.A., Lim W.A., Narlikar G.J., Almeida R. Nucleosome breathing and remodeling constrain CRISPR–Cas9 function. eLife. 2016; 5:e13450. 27130520 10.7554/eLife.13450 PMC4880442

[19] Horlbeck M.A. , Witkowsky L.B., Guglielmi B., Replogle J.M., Gilbert L.A., Villalta J.E., Torigoe S.E., Tjian R., Weissman J.S. Nucleosomes impede Cas9 access to DNA *in vivo* and *in vitro*. eLife. 2016; 5:e12677. 26987018 10.7554/eLife.12677 PMC4861601

[20] Hinz J.M. , Laughery M.F., Wyrick J.J. Nucleosomes selectively inhibit Cas9 off-target activity at a site located at the nucleosome edge. J. Biol. Chem. 2016; 291:24851–24856. 27756838 10.1074/jbc.C116.758706 PMC5122757

[21] McGinty R.K. , Tan S. Nucleosome structure and function. Chem. Rev. 2015; 115:2255–2273. 25495456 10.1021/cr500373h PMC4378457

[22] Makasheva K. , Bryan L.C., Anders C., Panikulam S., Jinek M., Fierz B. Multiplexed single-molecule experiments reveal nucleosome invasion dynamics of the Cas9 genome editor. J. Am. Chem. Soc. 2021; 143:16313–16319. 34597515 10.1021/jacs.1c06195 PMC8517959

[23] Hinz J.M. , LaugheryM.F., WyrickJ.J. Nucleosomes inhibit Cas9 endonuclease activity *in vitro*. Biochemistry. 2015; 54:7063–7066.2657993710.1021/acs.biochem.5b01108

[24] Yu X. , Buck M.J. Defining TP53 pioneering capabilities with competitive nucleosome binding assays. Genome Res. 2019; 29:107–115. 30409772 10.1101/gr.234104.117 PMC6314159

[25] Yu X. , Singh P.K., Tabrejee S., Sinha S., Buck M.J. ΔNp63 is a pioneer factor that binds inaccessible chromatin and elicits chromatin remodeling. Epigenetics Chromatin. 2021; 14:20. 33865440 10.1186/s13072-021-00394-8 PMC8053304

[26] Martin M. Cutadapt removes adapter sequences from high-throughput sequencing reads. EMBnet J. 2011; 17:10.

[27] Rognes T. , Flouri T., Nichols B., Quince C., Mahé F. VSEARCH: a versatile open source tool for metagenomics. PeerJ. 2016; 4:e2584. 27781170 10.7717/peerj.2584 PMC5075697

[28] Ekundayo B. , Richmond T.J., Schalch T. Capturing structural heterogeneity in chromatin fibers. J. Mol. Biol. 2017; 429:3031–3042. 28893533 10.1016/j.jmb.2017.09.002

[29] Anders C. , Niewoehner O., Duerst A., Jinek M. Structural basis of PAM-dependent target DNA recognition by the Cas9 endonuclease. Nature. 2014; 513:569–573. 25079318 10.1038/nature13579 PMC4176945

[30] Berman H.M. , Westbrook J., Feng Z., Gilliland G., Bhat T.N., Weissig H., Shindyalov I.N., Bourne P.E. The Protein Data Bank. Nucleic Acids Res. 2000; 28:235–242. 10592235 10.1093/nar/28.1.235 PMC102472

[31] Pettersen E.F. , Goddard T.D., Huang C.C., Meng E.C., Couch G.S., Croll T.I., Morris J.H., Ferrin T.E. UCSF ChimeraX: structure visualization for researchers, educators, and developers. Protein Sci. 2021; 30:70–82. 32881101 10.1002/pro.3943 PMC7737788

[32] Colasanti A.V. , Lu X.J., Olson W.K. Analyzing and building nucleic acid structures with 3DNA. J. Vis. Exp. 2013; 74:e4401. 10.3791/4401 PMC3667640 23644419

[33] Wang J. , Wang W., Kollman P.A., Case D.A. Automatic atom type and bond type perception in molecular mechanical calculations. J. Mol. Graph. Model. 2006; 25:247–260. 16458552 10.1016/j.jmgm.2005.12.005

[34] Pettersen E.F. , Goddard T.D., Huang C.C., Couch G.S., Greenblatt D.M., Meng E.C., Ferrin T.E. UCSF Chimera—a visualization system for exploratory research and analysis. J. Comput. Chem. 2004; 25:1605–1612. 15264254 10.1002/jcc.20084

[35] Bernard B. , Samudrala R. A generalized knowledge-based discriminatory function for biomolecular interactions. Proteins Struct. Funct. Bioinformatics. 2009; 76:115–128. 10.1002/prot.22323 PMC2891153 19127590

[36] Lowary P.T. , Widom J. New DNA sequence rules for high affinity binding to histone octamer and sequence-directed nucleosome positioning. J. Mol. Biol. 1998; 276:19–42. 9514715 10.1006/jmbi.1997.1494

[37] Doench J.G. , Fusi N., Sullender M., Hegde M., Vaimberg E.W., Donovan K.F., Smith I., Tothova Z., Wilen C., Orchard R. et al. Optimized sgRNA design to maximize activity and minimize off-target effects of CRISPR–Cas9. Nat. Biotechnol. 2016; 34:184–191. 26780180 10.1038/nbt.3437 PMC4744125

[38] Cruaud P. , Rasplus J.Y., Rodriguez L.J., Cruaud A. High-throughput sequencing of multiple amplicons for barcoding and integrative taxonomy. Sci. Rep. 2017; 7:41948. 28165046 10.1038/srep41948 PMC5292727

[39] Liu X. , Homma A., Sayadi J., Yang S., Ohashi J., Takumi T. Sequence features associated with the cleavage efficiency of CRISPR/Cas9 system. Sci. Rep. 2016; 6:19675. 26813419 10.1038/srep19675 PMC4728555

[40] Doench J.G. , Hartenian E., Graham D.B., Tothova Z., Hegde M., Smith I., Sullender M., Ebert B.L., Xavier R.J., Root D.E. Rational design of highly active sgRNAs for CRISPR–Cas9-mediated gene inactivation. Nat. Biotechnol. 2014; 32:1262–1267. 25184501 10.1038/nbt.3026 PMC4262738

[41] Nishimasu H. , Ran F.A., Hsu P.D., Konermann S., Shehata S.I., Dohmae N., Ishitani R., Zhang F., Nureki O. Crystal structure of Cas9 in complex with guide RNA and target DNA. Cell. 2014; 156:935–949. 24529477 10.1016/j.cell.2014.02.001 PMC4139937

[42] Barkal A.A. , Srinivasan S., Hashimoto T., Gifford D.K., Sherwood R.I. Cas9 functionally opens chromatin. PLoS One. 2016; 11:e0152683. 27031353 10.1371/journal.pone.0152683 PMC4816323

[43] Strohkendl I. , Saifuddin F.A., Gibson B.A., Rosen M.K., Russell R., Finkelstein I.J. Inhibition of CRISPR–Cas12a DNA targeting by nucleosomes and chromatin. Sci. Adv. 2021; 7:eabd6030. 33692102 10.1126/sciadv.abd6030 PMC7946368

[44] Anderson E.M. , Haupt A., Schiel J.A., Chou E., Machado H.B., Strezoska Z., Lenger S., McClelland S., Birmingham A., Vermeulen A. et al. Systematic analysis of CRISPR–Cas9 mismatch tolerance reveals low levels of off-target activity. J. Biotechnol. 2015; 211:56–65. 26189696 10.1016/j.jbiotec.2015.06.427

[45] Guintini L. , Charton R., Peyresaubes F., Thoma F., Conconi A. Nucleosome positioning, nucleotide excision repair and photoreactivation in *Saccharomyces cerevisiae*. DNA Repair. 2015; 36:98–104. 26429065 10.1016/j.dnarep.2015.09.012

[46] Liu X. *In vitro* chromatin templates to study nucleotide excision repair. DNA Repair. 2015; 36:68–76. 26531320 10.1016/j.dnarep.2015.09.026

[47] Meas R. , Wyrick J.J., Smerdon M.J. Nucleosomes regulate base excision repair in chromatin. Mutat. Res. Rev. Mutat. Res. 2019; 780:29–36. 31388331 10.1016/j.mrrev.2017.10.002 PMC6684245

[48] Yarrington R.M. , Verma S., Schwartz S., Trautman J.K., Carroll D. Nucleosomes inhibit target cleavage by CRISPR–Cas9 *in vivo*. Proc. Natl Acad. Sci. U.S.A. 2018; 115:9351–9358. 30201707 10.1073/pnas.1810062115 PMC6156633

[49] Anderson E.M. , Haupt A., Schiel J.A., Chou E., Machado H.B., Strezoska Ž., Lenger S., McClelland S., Birmingham A., Vermeulen A. et al. Systematic analysis of CRISPR–Cas9 mismatch tolerance reveals low levels of off-target activity. J. Biotechnol. 2015; 211:56–65. 26189696 10.1016/j.jbiotec.2015.06.427

[50] Fu B.X.H. , St Onge R.P., Fire A.Z., Smith J.D. Distinct patterns of Cas9 mismatch tolerance *in vitro* and *in vivo*. Nucleic Acids Res. 2016; 44:5365–5377. 27198218 10.1093/nar/gkw417 PMC4914125

[51] Meuleman W. , Muratov A., Rynes E., Halow J., Lee K., Bates D., Diegel M., Dunn D., Neri F., Teodosiadis A. et al. Index and biological spectrum of human DNase I hypersensitive sites. Nature. 2020; 584:244–251. 32728217 10.1038/s41586-020-2559-3 PMC7422677

[52] Asp P. , Blum R., Vethantham V., Parisi F., Micsinai M., Cheng J., Bowman C., Kluger Y., Dynlacht B.D. Genome-wide remodeling of the epigenetic landscape during myogenic differentiation. Proc. Natl Acad. Sci. U.S.A. 2011; 108:E149–E158. 21551099 10.1073/pnas.1102223108 PMC3107312

[53] Duan J. , Lu G., Xie Z., Lou M., Luo J., Guo L., Zhang Y. Genome-wide identification of CRISPR/Cas9 off-targets in human genome. Cell Res. 2014; 24:1009–1012. 24980957 10.1038/cr.2014.87 PMC4123298

[54] Brazelton V.A. Jr , Zarecor S., Wright D.A., Wang Y., Liu J., Chen K., Yang B., Lawrence-Dill C.J A quick guide to CRISPR sgRNA design tools. GM Crops Food. 2015; 6:266–276. 26745836 10.1080/21645698.2015.1137690 PMC5033207

[55] Xu H. , Xiao T., Chen C.H., Li W., Meyer C.A., Wu Q., Wu D., Cong L., Zhang F., Liu J.S. et al. Sequence determinants of improved CRISPR sgRNA design. Genome Res. 2015; 25:1147–1157. 26063738 10.1101/gr.191452.115 PMC4509999

[56] Singh R. , Kuscu C., Quinlan A., Qi Y., Adli M. Cas9–chromatin binding information enables more accurate CRISPR off-target prediction. Nucleic Acids Res. 2015; 43:e118. 26032770 10.1093/nar/gkv575 PMC4605288

[57] Heigwer F. , Kerr G., Boutros M. E-CRISP: fast CRISPR target site identification. Nat. Methods. 2014; 11:122–123. 24481216 10.1038/nmeth.2812

[58] Listgarten J. , Weinstein M., Kleinstiver B.P., Sousa A.A., Joung J.K., Crawford J., Gao K., Hoang L., Elibol M., Doench J.G. et al. Prediction of off-target activities for the end-to-end design of CRISPR guide RNAs. Nat. Biomed. Eng. 2018; 2:38–47. 29998038 10.1038/s41551-017-0178-6 PMC6037314

