# Supplementary material for: The impact of nucleosome structure on CRISPR/Cas9 fidelity
Source: Nucleic Acids Res. 2023 Feb 2;51(5):2333–44. doi: 10.1093/nar/gkad021 (PMC10018339; doi:10.1093/nar/gkad021)
Supplement: gkad021_Supplemental_File [file gkad021_supplemental_file.docx]

**Supplemental Tables and Figures**

**Supplemental Table 1. Target sequences in nucleosomes**

| Target | Sequence | PAM |
| --- | --- | --- |
| Forward target sequence 20 bp | 5'- GCAGGAACAGCGCCGGGGCG -3' | TGG |
| Reverse target sequence 20 bp | 5'- TCGGAGCAGCTGCTGCTACT -3' | GGG |
| Non-target sequence 20 bp | 5'- NNCTCGGTGATTGGCTCAGA -3' | AGG |

**Supplemental Table 2. Primer used for dephasing the nucleosome sequences**

| Amplicon Forward 1 | TCGTCGGCAGCGTCAGATGTGTATAAGAGACAGGATGGACCCTATACGCGGC |
| --- | --- |
| Amplicon Reverse 1 | GTCTCGTGGGCTCGGAGATGTGTATAAGAGACAGCCTCTGAACCTGGAACACTATCCGACTGGCA |
| Amplicon Forward 2 | TCGTCGGCAGCGTCAGATGTGTATAAGAGACAGAGCTCGAGAACGATGGACCCTATACGCGGC |
| Amplicon Reverse 2 | GTCTCGTGGGCTCGGAGATGTGTATAAGAGACAGGGAACACTATCCGACTGGCA |
| Amplicon Forward 3 | TCGTCGGCAGCGTCAGATGTGTATAAGAGACAGCTGAATGATGGACCCTATACGCGGC |
| Amplicon Reverse 3 | GTCTCGTGGGCTCGGAGATGTGTATAAGAGACAGTACTGTTCTGGAACACTATCCGACTGGCA |
| Amplicon Forward 4 | TCGTCGGCAGCGTCAGATGTGTATAAGAGACAGTCACTCTTGATGGACCCTATACGCGGC |
| Amplicon Reverse 4 | GTCTCGTGGGCTCGGAGATGTGTATAAGAGACAGATGGACGGGAACACTATCCGACTGGCA |

GATGGACCCTATACGCGGCCGCC**CTGGAGAATCCCGGTGCCGAGGCCGCTCAATTGGTCGTAGACAGCTCTAGCACCGCTTAAACGCACGTACGCGCTGTCCCCCGCGTTTTAACCGCCAAGGGGATTACTCCCTAGTCTCCAGGCACGTGTCAGATATATACATCCTGT**GCATGTATTGAACAGCGACCTTGCCGGTGCCAGTCGGATAGTGTTCC

**Supplemental Figure 1.** The Widom 601 sequence present in the GEMiNI-seq nucleosome library. The bolded sequence represents the 147 nucleotides involved in forming the Widom 601 nucleosome. The enlarged nucleotide indicates the Dyad position for Widom 601.


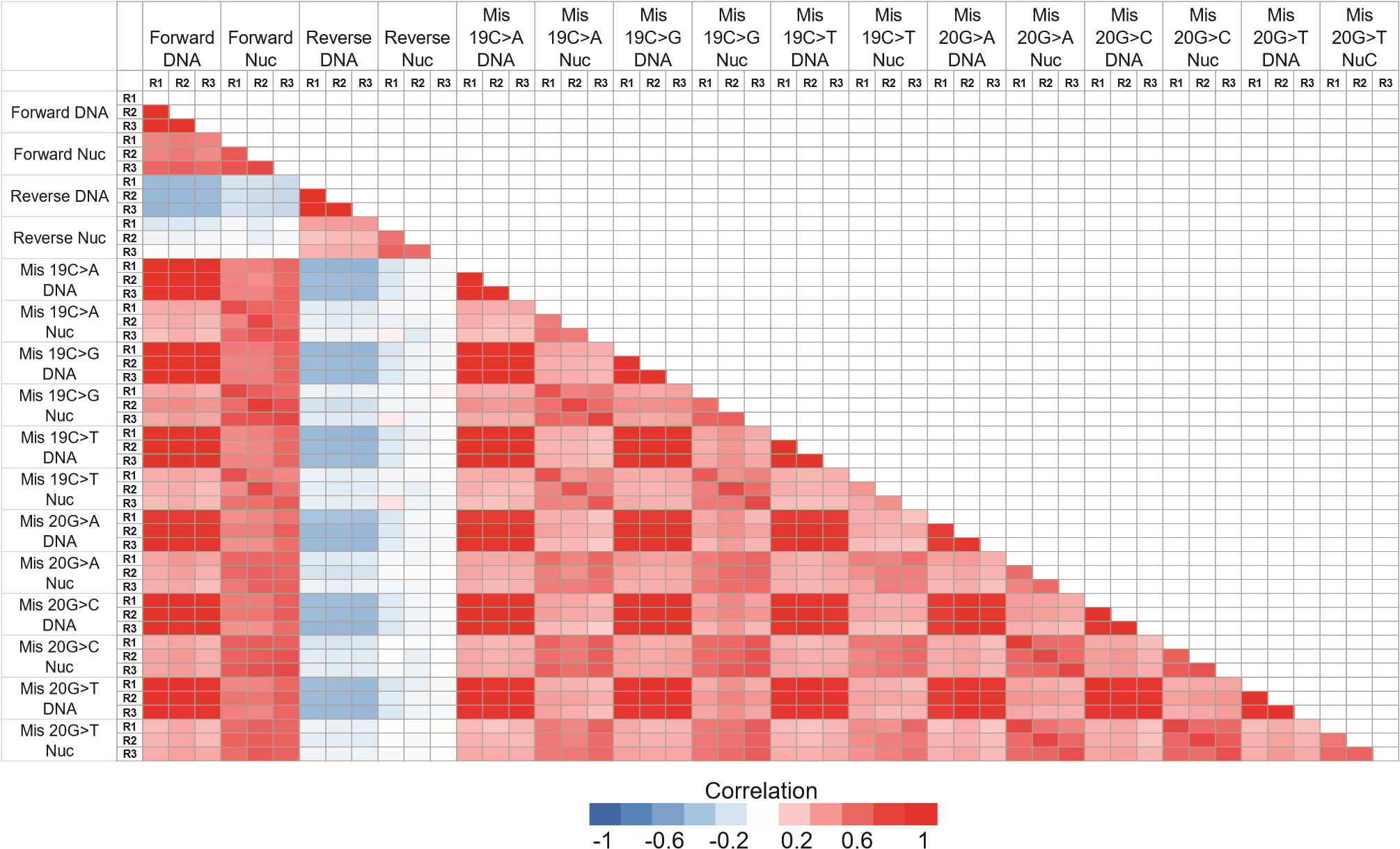


**Supplemental Figure 2.** The Pearson correlation for the protection for Cas9 across all experiments. The patterning shows a high similarity in the sample values within the replicates for each digestion, a similarity between the same sample types (Nucleosomes and naked DNA), and a similarity from similar used sgRNA sequences (the forward and mismatching sgRNA.)


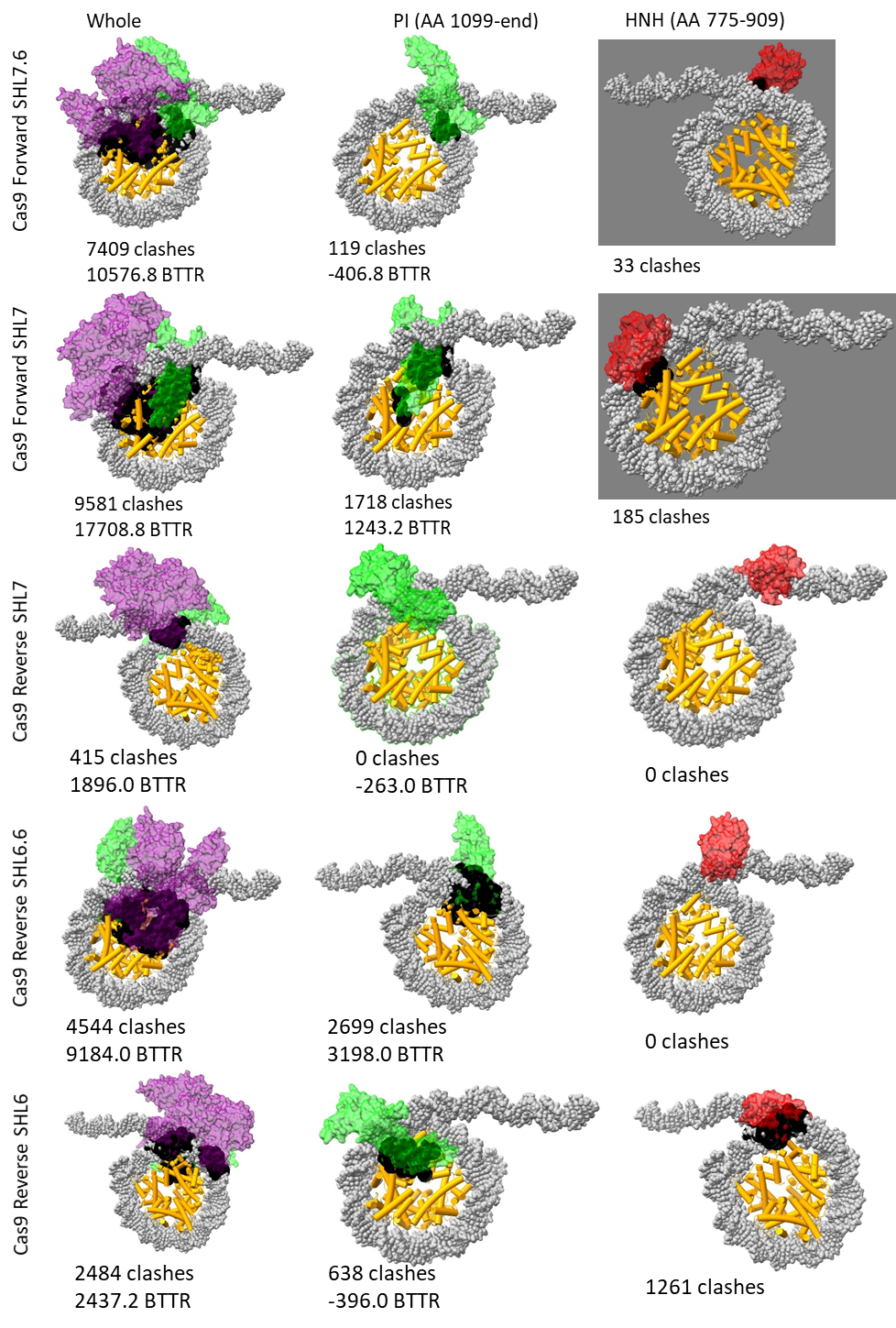


**Supplemental Figure 3.** The Cas9 endonuclease structure is imposed onto the various Forward (SHL 7 and 7.6) and Reverse target (SHL 6, 6.6, and 7). The Cas9 PI domain is green, the HNH domain is red, and the remaining Cas9 structure is purple. Black shading indicates the clashes present between the Cas9 and the nucleosome structure at the various imposed locations. The energy score for the entire Cas structure and the PI domain are determined by BTTR following minimization.


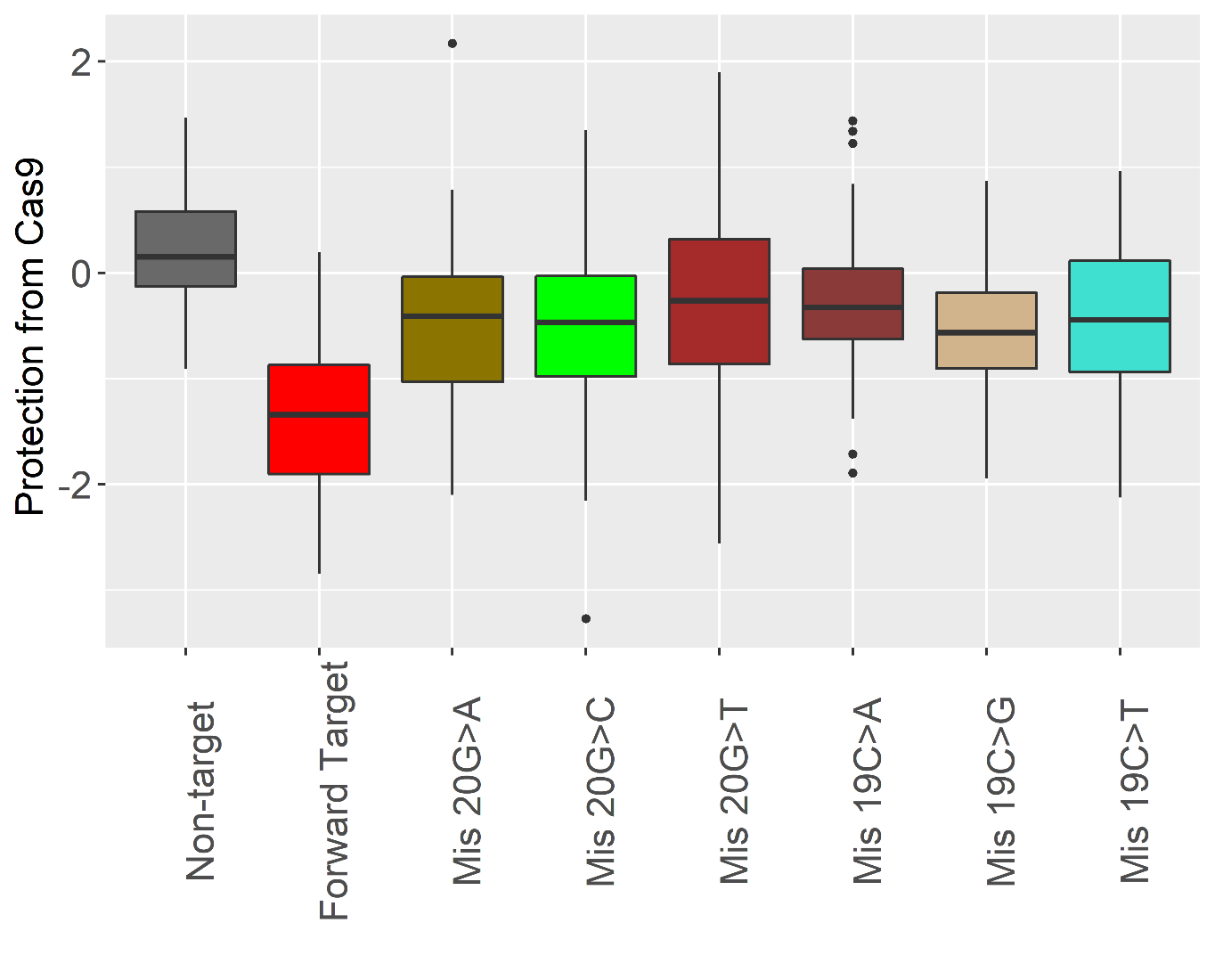


**Supplemental Figure 4.** The protection from Cas9 values for the forward target, non-target, and the mismatch targets within the nucleosome. The values graphed are for the nucleosomal region of SHL -4 to SHL 4.
